# Multiomics analysis demonstrated that TOPBP1 interacting checkpoint and Replication Regulator may serve as an immune-related biomarker indicative of poor prognosis in lung adenocarcinoma

**DOI:** 10.3389/fimmu.2025.1740279

**Published:** 2025-12-09

**Authors:** Chengbin Lin, Ziyi Chen, Wenbin Song, Long He, Hongyan Yu, Gaofeng Liang, Keyun Zhu, Jinxian He, Cangchang Shi

**Affiliations:** 1 Department of Thoracic Surgery, The Affiliated LiHuiLi Hospital of Ningbo University, Ningbo, Zhejiang, China; 2 Department of Thoracic Oncology, Tianjin Lung Cancer Center, Key Laboratory of Cancer Prevention and Therapy, Tianjin’s Clinical Research Center for Cancer, Tianjin Medical University Cancer Institute and Hospital, Tianjin Medical University, Tianjin, China; 3 Department of General Surgery, Tianjin Medical University General Hospital, Tianjin Key Laboratory of Precise Vascular Reconstruction and Organ Function Repair, Tianjin General Surgery Institute, Tianjin, China; 4 Department of General Surgery, Bishan Hospital Affiliated to Chongqing Medical University, Chongqing, China

**Keywords:** TICRR, lung adenocarcinoma, proliferation, invasion, immune evasion, therapeutic target

## Abstract

**Background:**

Lung adenocarcinoma (LUAD) persists as a leading contributor to global cancer-associated mortality. Identifying key oncogenic drivers is crucial for improving therapeutic strategies. This study aimed to investigate the role of TICRR in LUAD progression and its potential as a therapeutic target.

**Methods:**

Hub genes were screened through integration of DepMap CRISPR-Cas9 data, TCGA expression profiles, and survival analysis. TICRR expressions was assessed in LUAD tissues, adjacent controls, and cell lines by RT-qPCR and immunohistochemistry. Functional roles were examined using MTT, colony formation, transwell, wound healing, and EdU assays *in vitro*. Bioinformatics analyses, including GSEA, somatic mutation profiling, immune correlation, CMap drug sensitivity, and CT-based radiomics, were performed to explore mechanisms, therapeutic potential and clinical correlation.

**Results:**

TICRR expression was observed to be significantly elevated in LUAD tissues and cell lines, and its higher levels correlated with unfavorable patient outcomes and enrichment of malignant pathways, including EMT, E2F targets, and PI3K/AKT/mTOR signaling. High TICRR expression correlated with distinct somatic mutation patterns, increased tumor mutation burden, and elevated immune checkpoint expression. *In vitro*, TICRR knockdown suppressed Lung cancer progression. CMap analysis identified KU0063794 and CDK inhibitors as potential therapeutic agents targeting TICRR. Using a CT-based radiomics approach, the predictive model revealed a positive correlation between TICRR infiltration and radiological features in LUAD patients.

**Conclusion:**

TICRR functions as a critical oncogenic driver in LUAD, promoting proliferation, invasion, and immune evasion. Targeting TICRR may represent a novel strategy for personalized treatment of LUAD.

## Introduction

Lung cancer, is one of the most common malignancies in the world (1–3). Millions of people are newly diagnosed and die from the disease every year (4). Lung adenocarcinoma (LUAD) constitutes approximately 60% of newly diagnosed lung cancer cases, making it the most common subtype, with its incidence steadily increasing (5, 6). The primary therapeutic strategies for lung adenocarcinoma (LUAD) include surgical resection, cytotoxic chemotherapy, radiotherapy, molecularly targeted agents, and immunotherapy (7). Patients with early-stage lung adenocarcinoma usually exhibit a good prognosis (8, 9). However, some patients will develop advanced lung adenocarcinoma due to late diagnosis (10). Loss of surgical opportunities, limited treatments, and the drug resistance led to a poor prognosis in patients with advanced disease (11, 12). Therefore, the development of novel prognostic biomarker and therapeutic targets is urgent to improve clinical outcomes in patients with LUAD.

The TOPBP1-interacting checkpoint and replication regulator (TICRR), also referred to as C15orf42, FLJ41618, MGC45866, SLD3, or Treslin, was initially identified in Xenopus egg extracts. It functions in concert with TopBP1 during the Cdk2-dependent initiation phase of DNA replication, thereby contributing to the regulation of S-phase progression (13). Yu et al. discovered that TICRR promotes tumorigenesis by facilitating DNA replication (14). Furthermore, findings by He et al. revealed that TICRR overexpression is linked to adverse prognostic implications and altered patterns of immune cell infiltration within the tumor microenvironment of hepatocellular carcinoma (15). Chen et al. revealed that the overexpression of TICRR enhances disease aggressiveness and immune infiltration in cutaneous melanoma (16). Despite these studies confirming the association of TICRR with tumor development, its mechanism of action in tumors, particularly in LUAD, remains unclear. Our in-depth research on TICRR may provide new therapeutic strategies for lung cancer, thereby improving patient prognosis.

**Figure 1:**
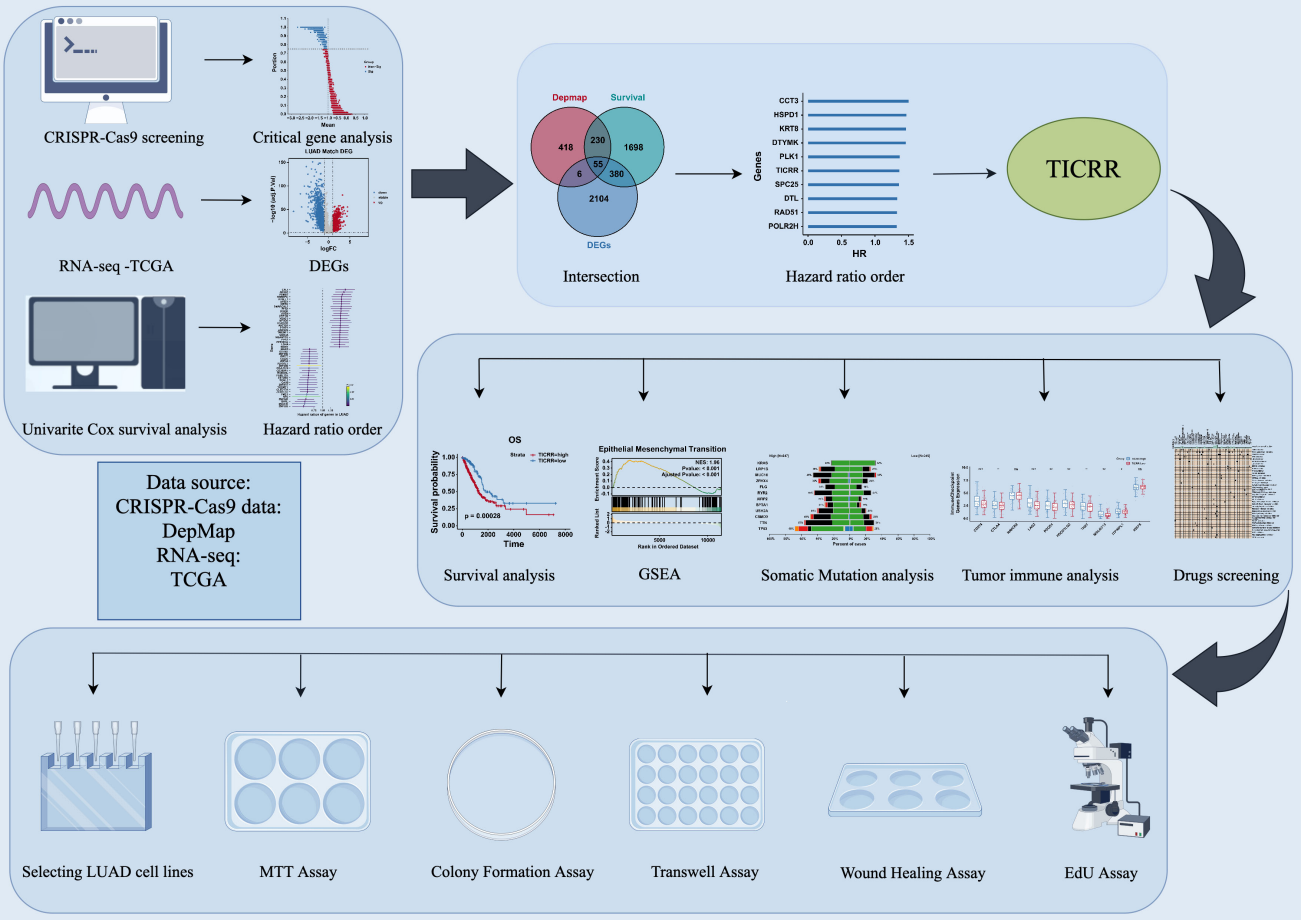
Schematic design of the study.

In the present study, we identified a significant association between TICRR expression and poor prognosis in patients with lung adenocarcinoma. Mechanistically, TICRR was found to facilitate tumor initiation and progression through activation of the PI3K/AKT signaling cascade. Functional suppression of TICRR significantly attenuated the proliferative and metastatic capacities of lung adenocarcinoma cells (**Figure 1**), highlighting TICRR as a promising therapeutic target in lung adenocarcinoma.

## Materials and methods

### Data acquisition and preprocessing

DepMap (The Cancer Dependency Map, https://depmap.org/portal/) is a research project that aims to generate comprehensive maps of cancer cell dependencies. This platform integrates extensive datasets from gene knockout experiments and gene expression profiling experiments across a large number of cancer cell lines, thereby facilitating researchers in gaining a deeper understanding of cancer mechanisms and developing novel therapeutic strategies. The CRISPR/Cas9 (Clustered Regularly Interspaced Short Palindromic Repeats-CRISPR Associated protein 9) system has evolved as an adaptive immune defense in prokaryotic organisms, providing protection against viral and plasmid invasion. It comprises the Cas9 nuclease and a single-guide RNA (sgRNA), which together form a ribonucleoprotein complex. Under the direction of the sgRNA, Cas9 specifically recognizes target DNA sequences via base-pair complementarity and induces site-specific double-strand breaks. By leveraging the CRISPR-Cas9 screening data from the DepMap database, further identification of key genes essential for the growth of lung adenocarcinoma cell lines can be achieved. The CERES score represents a normalized CRISPR-Cas9 gene knockout score, which serves as a metric for evaluating the level of gene dependency. A lower CERES score reflects a greater inhibitory impact on cell proliferation upon gene knockout. In this analysis, a CERES score of ≤ –1 was designated as the median threshold for common core essential genes. Genes meeting this criterion in at least 75% of lung adenocarcinoma cell lines were considered core essential for LUAD cell survival and proliferation.

### Identification of differentially expressed genes

Transcriptomic RNA-seq data of lung adenocarcinoma from The Cancer Genome Atlas (TCGA) was retrieved using the R package “TCGAbiolinks” (Version 2.33.0). Limma (Linear Models for Microarray Data), an R package widely applied in differential expression analysis of high-throughput datasets such as RNA-seq and ChIP-seq, was utilized for subsequent analyses. After removing duplicate samples and performing data normalization, the R package “limma” (Version 3.58.1) was employed to identify differentially expressed genes (DEGs) between lung adenocarcinoma tumor tissues and adjacent normal tissues. The thresholds for significance were set as a log2 fold change (logFC) of 1 and the adjusted P-value of 0.05. A volcano plot was used to visualize the distribution of 2545 DEGs.

### Univariate cox survival analysis and identification of hub genes

Using the patient survival data from the TCGA lung adenocarcinoma cohort, univariate Cox proportional hazards regression analyses were sequentially performed for all genes using the R package “survival” (Version 3.5-8). Genes with a p-value < 0.05 and a hazard ratio (HR) > 1 were defined as high-risk genes associated with prognosis and included in subsequent extraction of intersecting genes. Subsequently, researchers identified the set of key hub genes of interest by taking the intersection of three gene sets: core essential genes of lung adenocarcinoma cell lines from DepMap, differentially expressed genes in lung adenocarcinoma, and prognosis-related high-risk genes.

### Single-gene differential expression, survival analysis, and GSEA enrichment analysis

Transcriptomic data from The Cancer Genome Atlas (TCGA) were analyzed to assess the differential expression of TICRR between tumor and normal samples using a *t*-test, and the results were visualized as boxplots. TCGA-LUAD samples were subsequently divided into high- and low-expression groups according to the median TICRR expression level. Survival analyses were performed using the R packages “survminer” (v0.5.0) and “survival” (v3.5-8). Differentially expressed genes (DEGs) between the two TICRR expression groups were identified with R packages “limma” (v3.58.1). Genes showing statistically significant differences were ranked by log fold change (logFC) to generate an ordered list for Gene Set Enrichment Analysis (GSEA), which was conducted using “clusterProfiler”(v4.12.2) (17) and “GseaVis” (v0.0.5).

### Somatic mutation landscape and correlation analysis

Somatic mutation data of the TCGA-LUAD cohort were downloaded using the R package “TCGAbiolinks” (Version 2.33.0). The R package “maftools” (Version 2.20.0) was employed to construct MAF objects for somatic mutations in both the high-expression group and the low-expression group. Fisher’s exact test was performed on all genes across the two cohorts to identify differentially mutated genes and key gene pairs. GEPIA (http://gepia2.cancer-pku.cn/#index) provides a comprehensive and highly referenced resource for gene expression analysis, incorporating data from both TCGA and GTEx databases. Gene correlation analyses in tumor samples were conducted using this platform to investigate potential associations.

### Immune correlation analysis and immune cell infiltration

Immune checkpoint molecules act as inhibitory modulators of the immune system, critically contributing to self-tolerance, the prevention of autoimmunity, and the limitation of tissue damage by controlling immune response magnitude and duration. After obtaining transcriptomic counts and associated clinical data for lung adenocarcinoma from TCGA, the study focused on analyzing key genes implicated in immune regulation. Specifically, ITPRIPL1, SIGLEC15, TIGIT, CD274, HAVCR2, PDCD1, CTLA4, LAG3, PDCD1LG2, and IGSF8 were identified as pivotal genes in immune modulation. Based on transcriptomic data, the correlation between TICRR expression and immune checkpoint molecules was investigated. Subsequently, to ensure robust immune scoring, immunedeconv—a comprehensive R package integrating six algorithms for immune cell infiltration estimation—was employed. These algorithms include TIMER, xCell, MCP-counter, CIBERSORT, EPIC, and quanTIseq, each of which has been systematically benchmarked and exhibits distinct performance characteristics and advantages.

### Tumor mutation burden analysis and ssGSEA analysis

Tumor mutation burden (TMB), a metric attracting significant attention in immunotherapy research, was analyzed for its correlation with TICRR expression using Spearman’s correlation analysis. Genes involved in specific pathways were curated, and single-sample gene set enrichment analysis (ssGSEA) was performed using the “GSVA” package (Version 1.50.5). Finally, Spearman’s correlation analysis was employed to assess the relationship between gene expression levels and pathway enrichment scores.

### Drug sensitivity analysis

The Connectivity Map (CMap, https://clue.io/), a gene expression database established by the Cancer Systems Biology Consortium at the National Cancer Institute (NCI), serves as a platform for identifying potential therapeutic agents highly relevant to disease states. Leveraging the CMap tool, researchers submitted lists of upregulated and downregulated genes derived from expression profiling data to predict potential drug targets, thereby facilitating the discovery of candidate therapeutic agents targeting TICRR.

### Radiomics model establishment

Computed tomography (CT) imaging data, along with matched transcriptomic profiles from 29 lung adenocarcinoma patients, were retrieved from The Cancer Imaging Archive (TCIA). Radiomic feature extraction and analysis were performed using **3D Slicer** and its plugin platform, adhering to the Image Biomarker Standardization Initiative (IBSI) recommendations for ROI delineation and feature quantification. Tumor regions were manually segmented on each CT slice by experienced radiologists with more than five years of expertise, and all segmentations were reviewed by senior physicians. Any discrepancies were resolved through consensus with a third independent reviewer. After completion of layer-by-layer segmentation and three-dimensional tumor reconstruction, radiomic features were extracted. All CT images were resampled to a uniform voxel size to ensure consistency, and features were normalized using Z-score standardization. Cross-validation was performed using the glmnet R package to identify the optimal lambda (λ) for constructing a LASSO regression model aimed at predicting TICRR infiltration scores. Pearson correlation analysis was applied to select radiomic features significantly correlated with TICRR (*p* < 0.05). The model’s predictive capability was assessed via receiver operating characteristic (ROC) curve analysis.

### Immunohistochemistry

Tumor tissues embedded in paraffin were sectioned, deparaffinized, and rehydrated through a graded ethanol series. Antigen retrieval was carried out in citrate buffer, and endogenous peroxidase activity was blocked. Sections were incubated with TICRR-specific primary antibodies overnight at 4 °C, followed by incubation with HRP-conjugated secondary antibodies. Signal detection was performed using DAB, and nuclei were counterstained with hematoxylin. Stained slides were examined under an Olympus microscope, and ImageJ software was applied for quantitative analysis.

### RT-qPCR

Total RNA isolation from cultured cells was performed with TRIzol reagent (Invitrogen, USA), followed by reverse transcription into cDNA utilizing a PrimeScript RT kit (Takara, Japan). Quantitative PCR (qPCR) was performed using SYBR Green Master Mix (Applied Biosystems, USA) on a StepOnePlus Real-Time PCR system. GAPDH was used as the internal control, and relative gene expression levels were calculated using the 2^^−ΔΔCt^ method. Primer sequences are provided in the supplementary materials (**Supplementary Table S1**).

### Cell culture and transfection

The lung adenocarcinoma cell lines (HCC827, A549, H1975, H3122, H2228, H1299 and PC-9), and the normal lung cell line (BEAS-2B) were obtained from the Type Culture Collection of the Chinese Academy of Sciences. The cells were cultured in RPMI-1640 medium (Gibco, USA) containing 10% fetal bovine serum (FBS; PAN-Seratech) and 1% penicillin-streptomycin (PS; HyClone) in a 5% CO2 and humidified atmosphere at 37 °C. The siRNAs of TICRR constructs were synthesized by RiboBio (Guangzhou, China), sequences were listed in **Supplementary Table S2**. The protocol of cell transfection was conducted as previously described (18).

### MTT assay

Cell viability was evaluated using the MTT assay. Cells were plated in 96-well plates and cultured for the specified durations. After a 4-hour incubation with MTT solution (5 mg/mL), formazan crystals were solubilized in DMSO, and the absorbance at 570 nm was recorded using a microplate reader.

### Colony formation assay

For colony formation assays, 800 cells were plated per well in 6-well plates and maintained under standard culture conditions for 10–14 days until colonies became visible. Cells were fixed with 4% paraformaldehyde, stained with crystal violet, and colonies comprising more than 50 cells were quantified using a light microscope.

### Transwell assay

Transwell assays were conducted to assess cell migratory and invasive capabilities using 8-μm pore membranes (Corning, USA). For migration assays, serum-starved cells were placed in the upper chamber, and the lower chamber contained medium with 10% FBS as a chemoattractant. For invasion assays, membranes were coated with Matrigel (BD Biosciences) before seeding. After incubation, non-migratory or non-invasive cells were removed, and the remaining cells on the lower surface were fixed, stained with crystal violet, and counted under a microscope.

### Wound healing assay

Cells were grown in 6-well plates until reaching near confluence, after which a linear scratch was made across the monolayer with a sterile pipette tip. Floating cells were gently washed away, and cultures were maintained in serum-free medium. To document wound healing progression, images of the scratch wound were acquired at 0 hours and at designated intervals thereafter using an inverted microscope. The migration rate was quantified with ImageJ software.

### EdU assay

Cells were seeded into 96-well plates, incubated with EdU reagent (Ribobio, China) for the indicated time, and then fixed with 4% paraformaldehyde. After permeabilization, incorporated EdU was detected following the manufacturer’s protocol, and nuclei were counterstained with DAPI. Fluorescent images were acquired under a fluorescence microscope, and the percentage of EdU-positive cells was calculated.

## Results

### Identification of hub gene set

**Figure 2:**
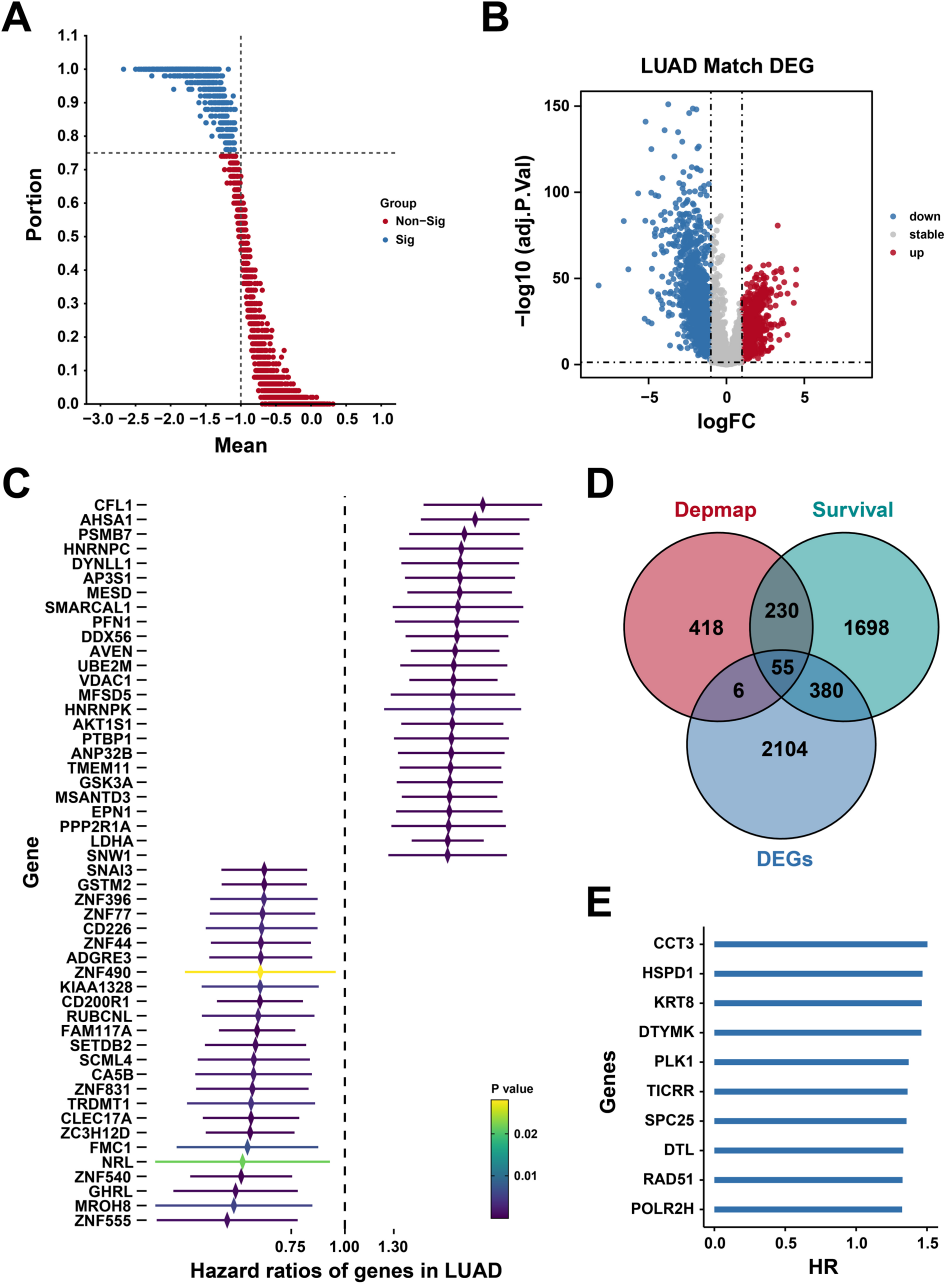
Identification of hub gene set. **(A)** The CRISPR-CAS9 data screening in lung adenocarcinoma. **(B)** The upregulated genes and downregulated genes in LUAD. **(C)** The univariate COX survival analysis of each gene. **(D)** The Venn maps of differentially expressed genes and high-risk genes. **(E)** The top 10 genes ranked by HR displayed.

To uncover key regulators of lung adenocarcinoma, we interrogated CRISPR-Cas9 dependency data from DepMap, identifying 709 genes indispensable for the growth of LUAD cell lines (**Figure 2A**). Parallel differential expression analysis in TCGA-LUAD samples revealed 888 upregulated and 1,657 downregulated genes relative to normal tissues (**Figure 2B**), establishing a comprehensive gene set for downstream analyses. By integrating the expression profile data and clinical data from the TCGA-LUAD cohort, we performed univariate COX survival analysis for each gene and visualized the top 25 and bottom 25 genes ranked by Hazard Ratio (HR) (**Figure 2C**). Taking the intersection of genes identified from CRISPR-Cas9 screening, differentially expressed genes in lung adenocarcinoma, and high-risk genes associated with survival, a total of 55 hub genes were obtained (**Figure 2D**), with the top 10 genes ranked by HR displayed (**Figure 2E**).

### TICRR is highly expressed in lung adenocarcinoma and associated with malignant tumor pathways

**Figure 3:**
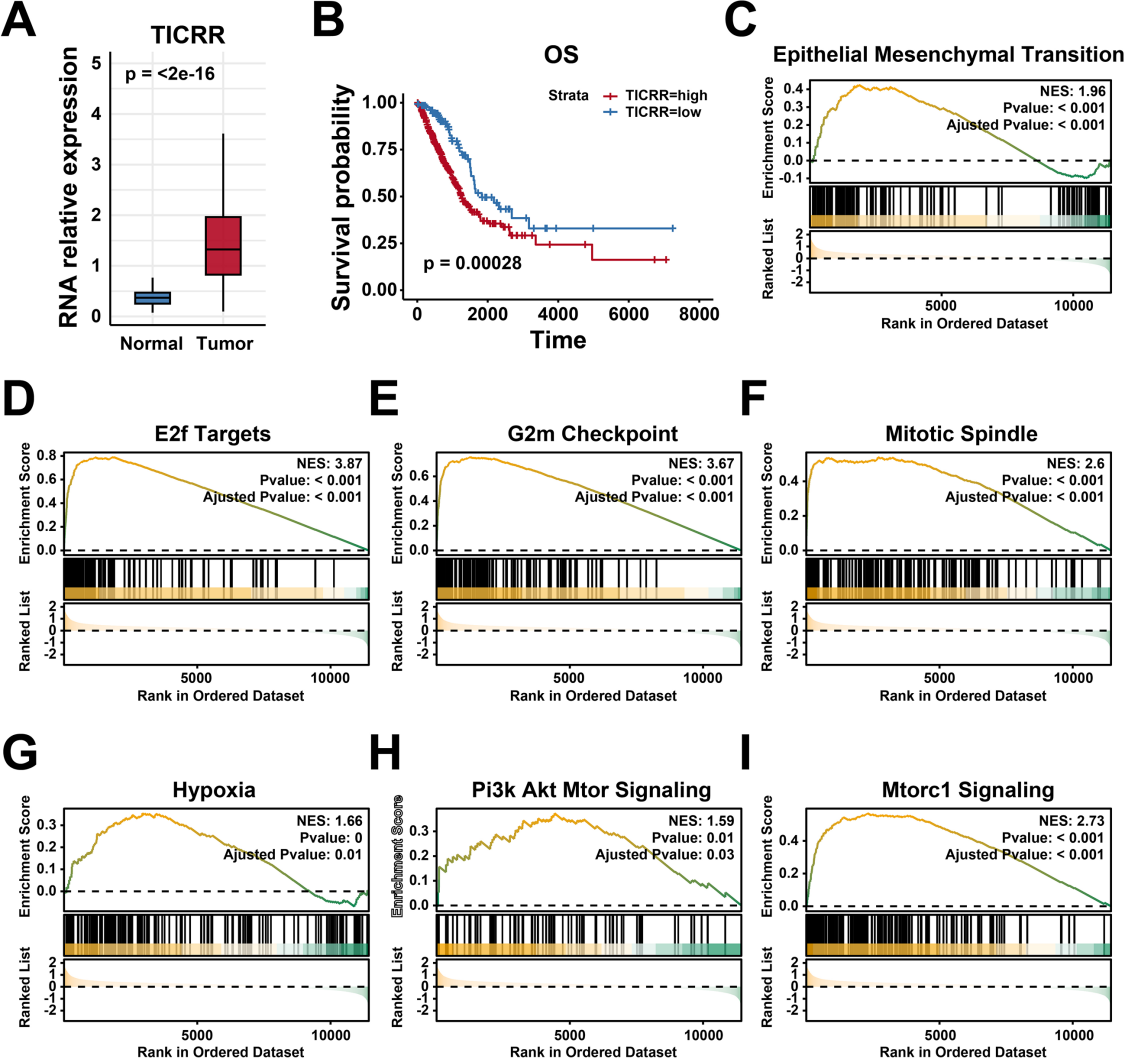
TICRR is highly expressed in lung adenocarcinoma and associated with malignant tumor pathways. **(A)** The expression of TICRR in TCGA-LUAD cohort. **(B)** Survival analysis of TICRR expression in LUAD patients. GSEA analysis of TICRR, including EMT **(C)**, E2F target pathway **(D)**, G2M checkpoint pathway **(E)**, Mitotic Spindle **(F)**, Hypoxia **(G)**, PI3K-AKT-mTOR signaling pathway **(H)** and mTORC1 signaling pathway **(I)**.

Analysis of the TCGA-LUAD cohort demonstrated a significant upregulation of TICRR in tumor tissues, corroborating our previous findings (**Figure 3A**). Kaplan–Meier survival analysis revealed that elevated TICRR expression was associated with poorer patient outcomes (**Figure 3B**). Gene Set Enrichment Analysis (GSEA) showed that samples with high TICRR expression were significantly enriched in pathways linked to tumor malignancy, including epithelial-mesenchymal transition (EMT) (**Figure 3C**), E2F target genes (**Figure 3D**), G2M checkpoint (**Figure 3E**), mitotic spindle (**Figure 3F**), hypoxia (**Figure 3G**), PI3K-AKT-mTOR signaling (**Figure 3H**), and mTORC1 signaling (**Figure 3I**). Collectively, the TICRR gene is closely associated with adverse patient prognosis and malignant tumor phenotypes.

### TICRR and somatic mutation characteristics

**Figure 4:**
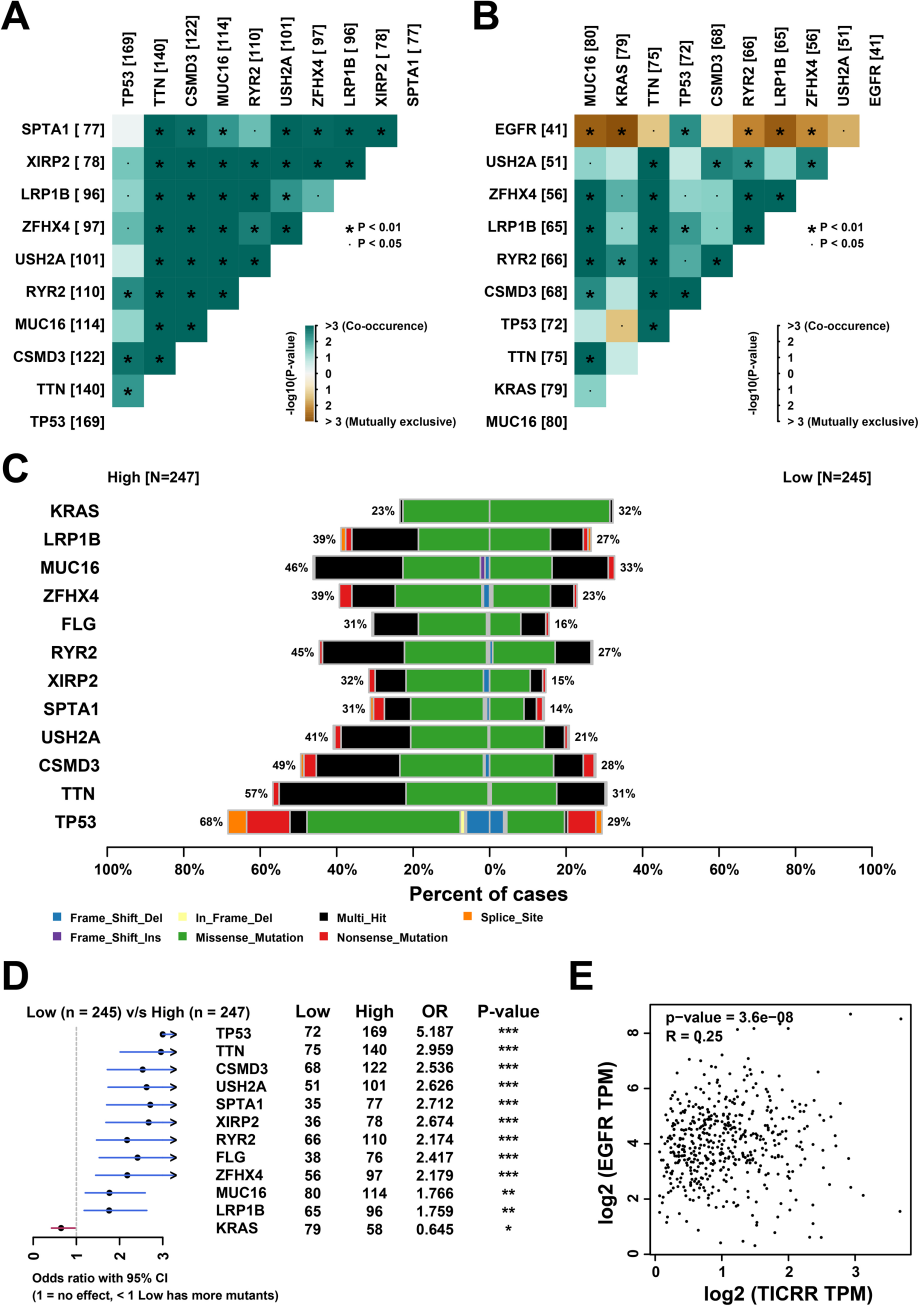
TICRR and somatic mutation characteristics. The somatic mutation analysis in both TICRR high-expression groups **(A)** and low-expression groups **(B)**. **(C)** The distribution and proportion of different mutation types of genes in TICRR high-expression group and low-expression group. **(D)** Forest plot in somatic single nucleotide variants (SNVs) between patients with high and low expression of TICRR. OR > 1 indicates a higher frequency of SNVs in the high TICRR group, OR < 1 indicates a higher frequency in the low infiltration group. *p < 0.05, **p < 0.01, ***p < 0.001. **(E)** The correlation between TICRR and EGFR expression.

To further investigate the association between TICRR and somatic mutation characteristics, we analyzed the somatic mutation profiles in both TICRR high-expression and low-expression groups. Researchers observed a co-mutation pattern of somatic mutations in the TICRR high-expression group (**Figure 4A**), whereas a partially mutually exclusive mutation pattern was noted in the TICRR low-expression group (**Figure 4B**), particularly for the EGFR gene. The frequency of gene mutations was relatively lower in the low-expression group, while genes such as TP53, TTN, and CSMD3 exhibited higher mutation frequencies in the high-expression group. Additionally, the primary mutation types varied among different genes (**Figure 4C**). In the high-expression group, genes including TP53, TTN, CSMD3, USH2A, SPTA1, XIRP2, RYR2, FLG, ZFHX4, MUC16, and LRP1B showed higher mutation rates, whereas KRAS exhibited a lower mutation rate (**Figure 4D**). Interestingly, based on the mutually exclusive mutation pattern between EGFR and low TICRR expression, we found a positive correlation between the expression levels of TICRR and EGFR (**Figure 4E**). These results suggest that mutation characteristics of different genes vary between the high- and low-expression groups, and high TICRR expression may be associated with a higher number of mutations.

### TICRR enhances tumor immune evasion and immune suppression

**Figure 5:**
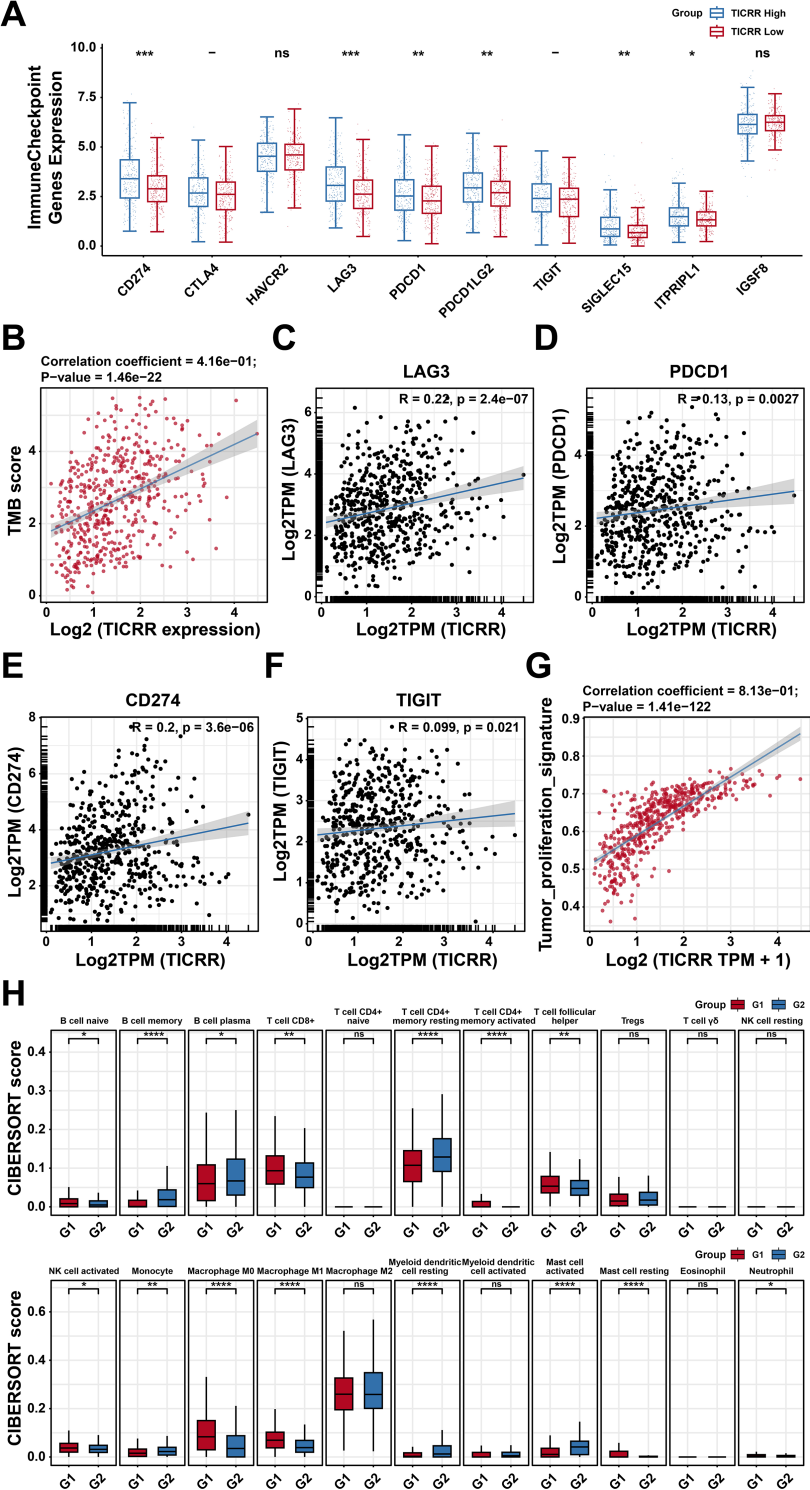
TICRR enhances tumor immune evasion and immune suppression. **(A)** The differences in the expression of immune checkpoints between the high-expression group and the low-expression group of TICRR. **(B)** The correlation between TICRR expression and TMB score. **(C-F)**. The correlation between the expression of TICRR and different immune checkpoints. **(G)** The correlation between TICRR expression and tumor proliferation characteristics score. **(H)** Comparison of different immune cell infiltration situations in the high-expression group and the low-expression group of TICRR. *p < 0.05, **p < 0.01, ***p < 0.001

To elucidate the potential role of TICRR in tumor immunity, we assessed its relationship with key immune checkpoint molecules. The TICRR high-expression group displayed significantly elevated levels of CD274, LAG3, PDCD1, PDCD1LG2, SIGLEC15, and ITPRIPL1 compared with the low-expression group (**Figure 5A**). Additionally, tumor mutation burden (TMB) analysis revealed that high TICRR expression was associated with increased TMB (**Figure 5B**). Correlation analyses further demonstrated positive associations between TICRR and immune checkpoints including LAG3, PDCD1, CD274, and TIGIT (**Figures 5C-F**). Interestingly, TICRR also showed a positive correlation with tumor proliferation characteristics (**Figure 5G**). Based on the TICRR expression, we conducted an analysis of immune cell infiltration. (**Figure 5H**). These findings suggest that TICRR may enhance tumor immune evasion and immune suppression through multiple pathways.

### Potential therapeutic drugs targeting TICRR

**Figure 6:**
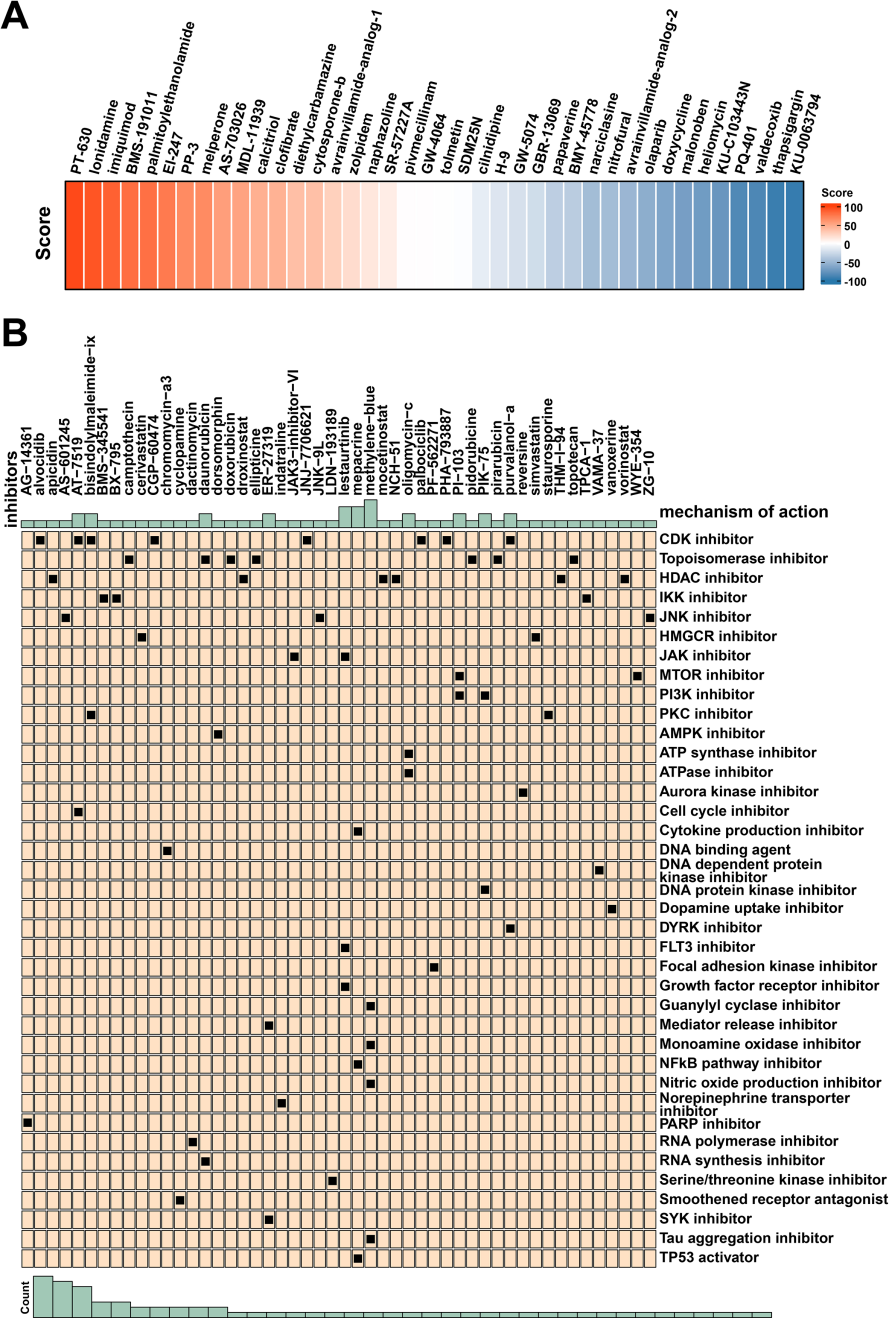
Potential therapeutic drugs targeting TICRR. **(A)** The CMAP drug connectivity score based on the expression of TICRR. **(B)** The potential drugs identified for high-TICRR patients.

Through Connectivity Map (CMap) drug sensitivity analysis, researchers identified KU0063794 as the most promising therapeutic agent for TICRR, indicating that TICRR may be sensitive to this potent and specific mTOR inhibitor (**Figure 6A**). Additionally, high TICRR expression may be associated with increased sensitivity to CDK inhibitors, suggesting that TICRR may play a role in CDK-related pathways (**Figure 6B**). These findings could assist clinicians in selecting more effective treatment regimens based on the TICRR expression levels of patients, thereby improving therapeutic efficacy. The CMap drug sensitivity result chart illustrates the association between TICRR expression levels and drug sensitivity. Analyzing the differences in sensitivity to various drugs and their mechanisms of action can provide guidance for personalized treatment and offer clues for further mechanistic studies and drug development.

### TICRR is up-regulated in LUAD tissues and cells

**Figure 7:**
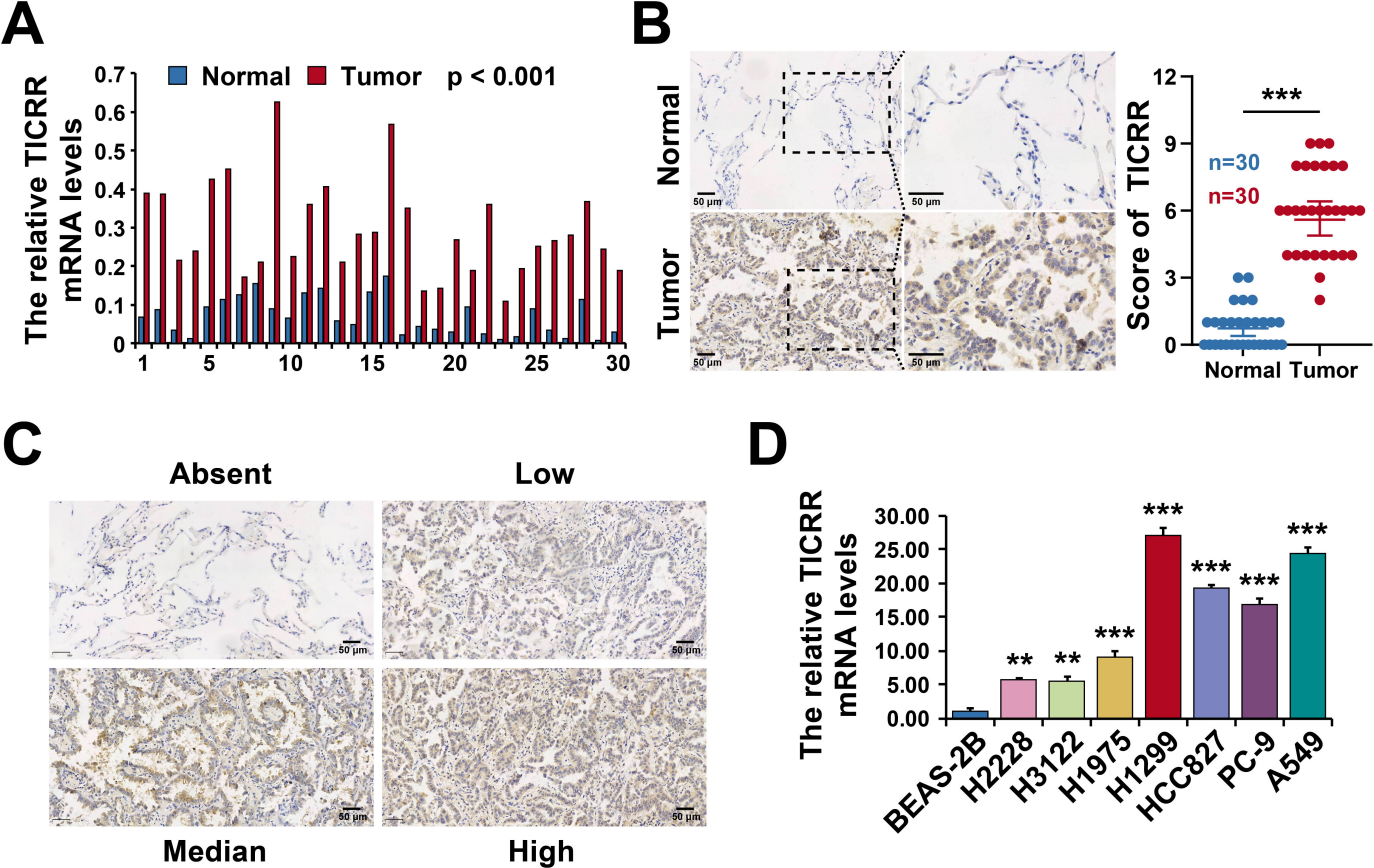
TICRR is up-regulated in LUAD tissues and cells. **(A)** The relative TICRR mRNA levels in LUAD tissues detected by RT-qPCR. **(B)** The TICRR expression levels in LUAD tissues and paired normal tissues detected by IHC. **(C)** Representative images of staining at different scoring levels. **(D)** The relative TICRR mRNA levels in Lung cancer cells and normal lung epithelial cell line detected by RT-qPCR. *p < 0.05, **p < 0.01, ***p < 0.001

To investigate the expression of TICRR in LUAD, we collected 30 pairs of LUAD tissues and adjacent normal tissues. RT-qPCR analysis revealed that TICRR mRNA levels were significantly elevated in tumor tissues compared to matched controls (**Figure 7A**). Consistently, immunohistochemical staining of tissue sections demonstrated higher TICRR protein expression in lung cancer (**Figure 7B**). Representative images of staining at different scoring levels are shown as **Figure 7C**. We further assessed TICRR expression in a panel of lung cancer cell lines (H2228, H3122, H1975, H1299, HCC827, PC-9, and A549) and compared them with the normal lung epithelial cell line BEAS-2B. RT-qPCR showed markedly higher TICRR mRNA expression in cancer cells, with the highest level observed in H1299 cells (**Figure 7D**). Collectively, these findings indicate that TICRR is significantly upregulated in both LUAD tissues and cell lines, suggesting its potential role as an oncogenic factor.

### Downregulation of TICRR inhibits LUAD progression

**Figure 8:**
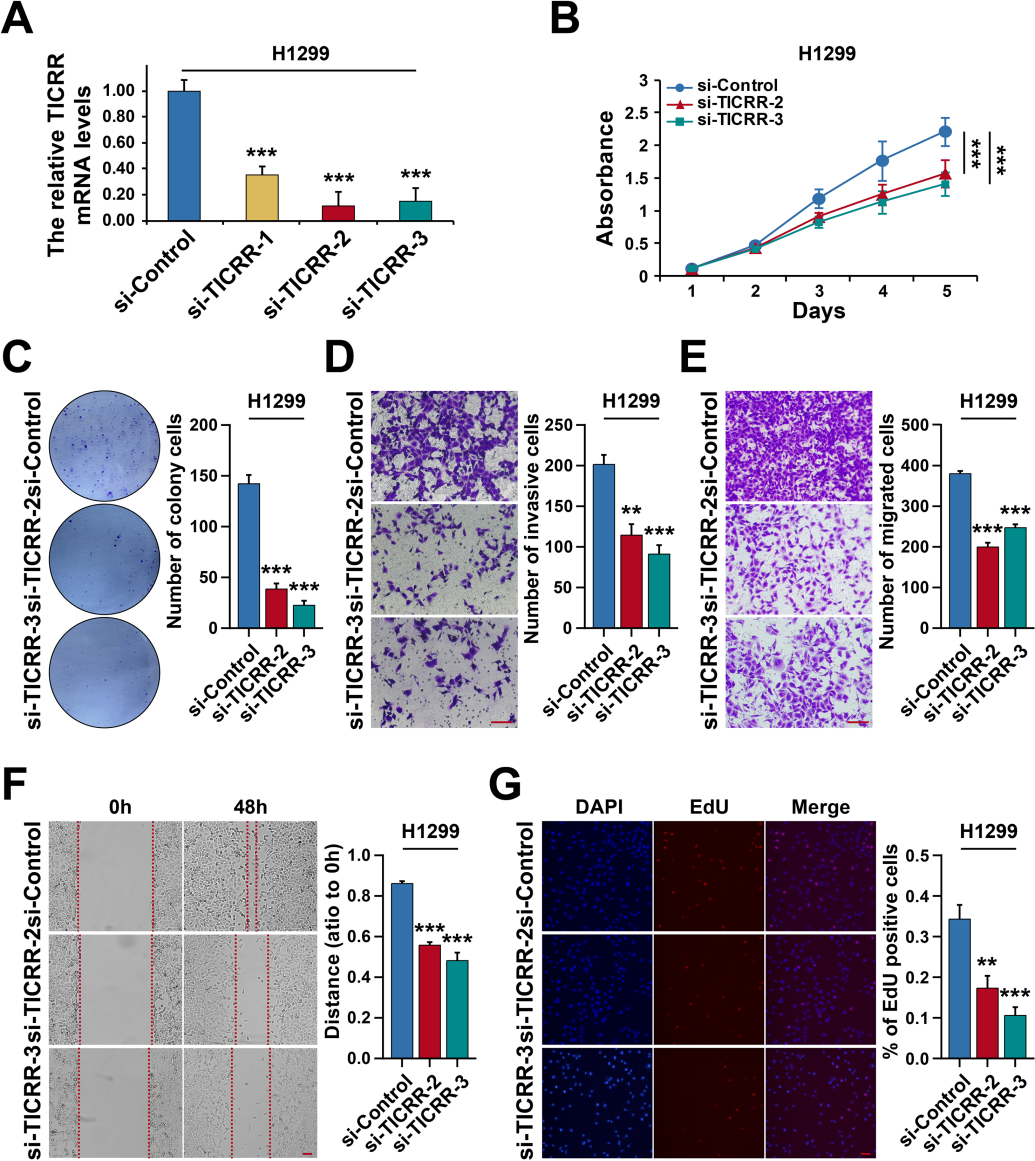
Downregulation of TICRR inhibits LUAD Progression in H1299 cells. **(A)** Verification of TICRR knockdown efficiency by RT-qPCR. **(B)** The cell proliferation of TICRR-deleted H1299 cells detected by MTT assay. **(C)** Colony formation assay in TICRR-deleted H1299 cells. The cell invasion and migration assay of TICRR-deleted H1299 cells with **(D)** or without Matrigel **(E)**. **(F)** wound healing assays of TICRR-deleted H1299 cells. **(G)** EdU assay in TICRR-deleted H1299 cells. *p < 0.05, **p < 0.01, ***p < 0.001.

We next investigated the functional role of TICRR in LUAD cells through a series of *in vitro* assays. In H1299 cells, which exhibit relatively high baseline TICRR expression, transient knockdown was achieved by transfecting small interfering RNAs targeting TICRR. RT-qPCR confirmed efficient suppression, and si-TICRR-2 and si-TICRR-3, which showed the highest knockdown efficiency (**Figure 8A**), were selected for subsequent experiments. MTT assays demonstrated that TICRR silencing significantly inhibited cell proliferation (**Figure 8B**). Consistently, colony formation assays revealed that downregulation of TICRR markedly reduced both the number and size of colonies (**Figure 8C**). Transwell assays, performed with or without Matrigel, showed that TICRR knockdown suppressed cell invasion (**Figure 8D**) and migration (**Figure 8E**), while wound healing assays further confirmed impaired migratory ability (**Figure 8F**). In addition, EdU staining indicated a significant reduction in the proportion of EdU-positive cells following TICRR silencing, supporting the conclusion that TICRR promotes proliferation (**Figure 8G**). These functional assays were replicated in A549 cells, yielding consistent results (**Supplementary Figure S1A-G**). Collectively, these findings demonstrate that TICRR knockdown significantly suppresses the proliferation, invasion, and migration of lung cancer cells, highlighting its potential role as an oncogenic factor.

### The infiltration abundance of TICRR in LUAD can be measured using a radiomics model based on CT imaging.

**Figure 9:**
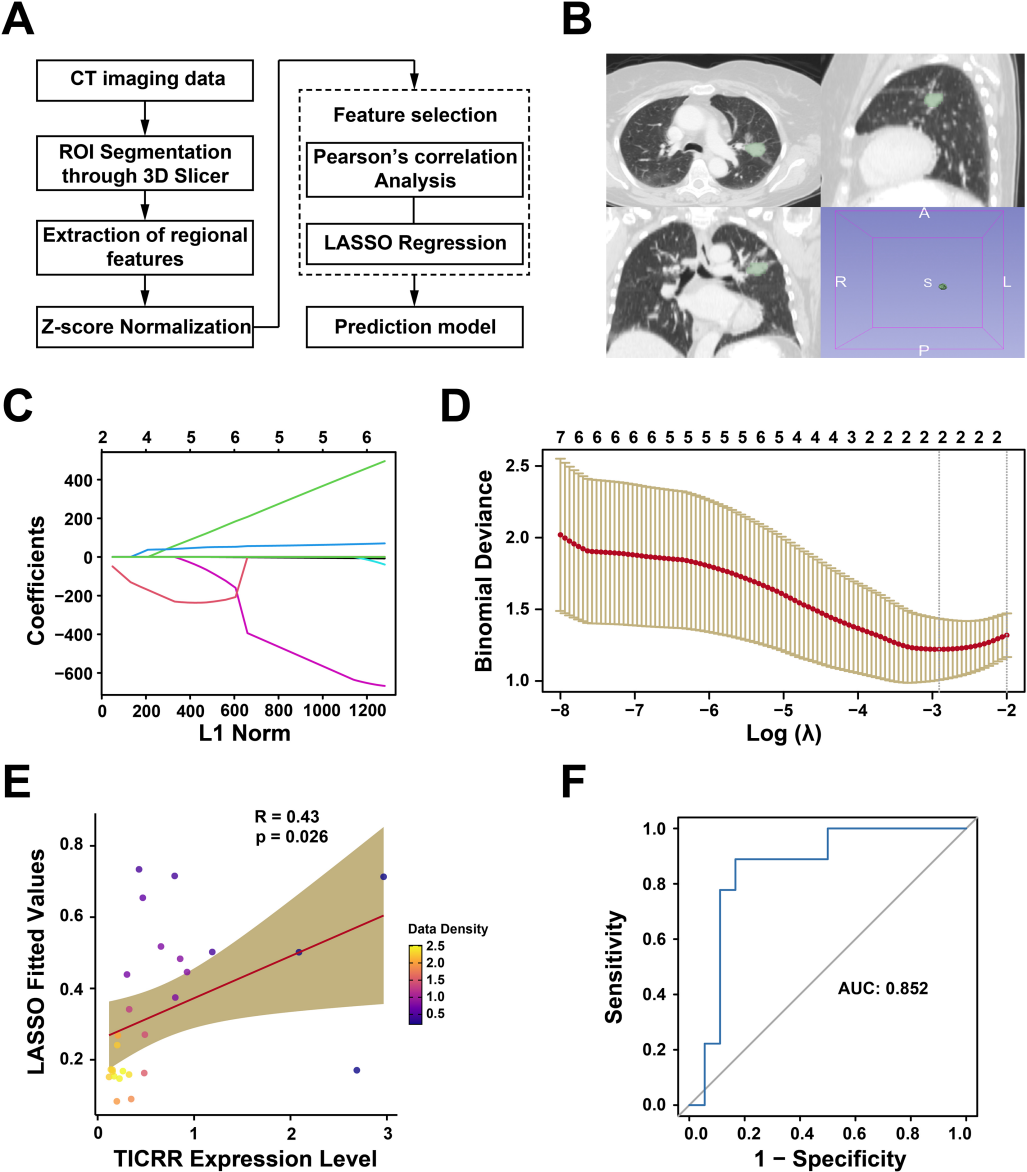
The infiltration abundance of TICRR in LUAD can be measured using a radiomics model based on CT imaging. **(A)** Schematic diagram illustrating the radiomics analysis process. **(B)** Example of segmenting Regions of Interest (ROIs) using 3D Slicer software. **(C)** In the LASSO regression analysis, the coefficient distribution of the variables is depicted. Each curve corresponds to radiomic features that have been filtered using Pearson correlation. The y-axis shows the coefficients associated with these features in the LASSO regression. The value at the top indicates the number of radiomic features that were selected via Pearson correlation and subsequently incorporated into the LASSO regression model. **(D)** Plot of Selection Operator (LASSO) regression. **(E)** The Pearson correlation coefficient was computed between the z-score normalized abundance of TICRR and the fitted values obtained from the linear regression radiomics model. **(F)** The receiver operating characteristic (ROC) curve demonstrating the model’s capability to differentiate the levels of TICRR abundance.

To investigate the clinical applicability of TICRR, we implemented a non-invasive radiomics-based strategy to estimate TICRR infiltration levels (**Figure 9A**). Twenty-nine LUAD patients from the TCIA database were included, with regions of interest (ROIs) manually delineated in 3D Slicer and subsequently verified and refined by senior clinicians to ensure precise tumor segmentation (**Figure 9B**). Pearson correlation analysis identified seven radiomic features associated with TICRR abundance. LASSO regression was then applied for dimensionality reduction (**Figures 9C, D**), resulting in two features selected to construct the final radiomics linear regression model. TICRR abundance was strongly correlated with radiomics scores (R = 0.42, *p* = 0.026, **Figure 9E**), and ROC analysis indicated that radiomics scores could effectively predict TICRR infiltration (AUC = 0.852, **Figure 9F**). These findings demonstrate a novel non-invasive approach for assessing TICRR infiltration in LUAD patients.

## Discussion

In this study, we comprehensively investigated the oncogenic role of TICRR in lung adenocarcinoma (LUAD). Through integrated bioinformatics analysis, we identified TICRR as a hub gene associated with poor prognosis. Consistently, experimental validation revealed that TICRR was significantly upregulated in LUAD tissues and cell lines compared to normal controls. Importantly, functional assays demonstrated that TICRR promotes tumor proliferation, invasion, and migration. These findings strongly support the notion that TICRR acts as an oncogenic driver in LUAD. Besides, our radiomics analysis further revealed that TICRR infiltration levels in LUAD could be effectively estimated through non-invasive CT-based imaging features. The strong correlation between TICRR abundance and radiomics scores suggests that radiogenomic signatures may reflect the underlying molecular phenotype of TICRR-driven tumors. These findings highlight the potential of integrating imaging biomarkers with transcriptomic data to facilitate precise, non-invasive assessment of tumor biological behavior.

Mechanistically, TICRR’s biological role is consistent with regulation of DNA replication initiation and S-phase progression; depletion of TICRR impairs both origin firing and fork progression, induces DNA damage responses and cell-cycle arrest, and thereby suppresses tumor growth (14). Consistent with this, broader literature links TICRR overexpression to adverse outcomes in other cancers (e.g., endometrial and papillary renal cell carcinoma) and to cell-cycle/replication control, underscoring the generalizability of our findings (19, 20). Typically, the progression of malignant tumors is associated with the aberrant activation or suppression of various signaling pathways (21–23). Our pathway analyses and experimental data linked TICRR to oncogenic signaling axes beyond canonical replication control. High TICRR expression correlated with activation of EMT, E2F, G2/M checkpoint and PI3K/AKT/mTOR pathways (16), and TICRR knockdown reduced malignant phenotypes—findings that align with recent studies showing TICRR can potentiate PI3K/AKT/mTOR signaling and thereby enhance tumor aggressiveness and immune infiltration in other tumor contexts (24). Besides, preliminary functional data support a model wherein TICRR promotes lung cancer pathogenesis by modulating the PI3K/AKT/mTOR pathway and its downstream network. This offers a plausible mechanism for the observed phenotypes. However, the precise regulatory mechanism remains to be defined and is a key objective for future work.

However, our research has some limitations. First, our primary discovery relied on retrospective public datasets (TCGA, DepMap) and in silico inference; although we performed laboratory validation, prospective validation in larger multi-center cohorts is required to establish TICRR’s prognostic utility and clinical predictive value. Second, while our functional data support a role for TICRR in promoting proliferation, invasion and metastasis, the precise molecular links between TICRR, PI3K/AKT/mTOR activation, immune modulation, and specific mutational contexts remain to be dissected, as well as the validation *in vivo*. Recent cancer-type–specific studies reinforce the heterogeneity of TICRR’s downstream effects and underscore the need for tumor-specific mechanistic work and biomarker development.

In summary, our integrated multi-omics analysis and functional validation establish TICRR as an oncogenic driver in LUAD that promotes tumor proliferation, invasion, and metastatic dissemination. TICRR expression is further linked to distinct somatic mutation profiles and immune microenvironment features, while also revealing potential therapeutic vulnerabilities. These findings nominate TICRR as a promising therapeutic target worthy of further investigation for improving clinical outcomes in LUAD patients.

## Data Availability

The original contributions presented in the study are included in the article/**Supplementary Material**. Further inquiries can be directed to the corresponding authors.

## Supplementary material

Supplementary Figure 1 Downregulation of TICRR inhibits LUAD Progression in A549 cells. **A.** Verification of TICRR knockdown efficiency by RT-qPCR. **B**. The cell proliferation of TICRR-deleted A549 cells detected by MTT assay. **C.** Colony formation assay in TICRR-deleted A549 cells. The cell invasion and migration assay of TICRR-deleted A549 cells with (**D**) or without Matrigel (**E**). **F.** wound healing assays of TICRR-deleted A549 cells. **G.** EdU assay in TICRR-deleted A549 cells. *p < 0.05, **p < 0.01, ***p < 0.001.

Supplementary Figure 2 **A.** Expression of TICRR in LUAD based on patient’s race. **B**. The related statistical significance.

Supplementary Figure 3 **A.** Expression of TICRR across TCGA cancers. **B**. The association between TICRR and G2M checkpoint.
