# Supplementary material for: Multiomics analysis demonstrated that TOPBP1 interacting checkpoint and Replication Regulator may serve as an immune-related biomarker indicative of poor prognosis in lung adenocarcinoma
Source: Front Immunol. 2025 Dec 9;16:1740279. doi: 10.3389/fimmu.2025.1740279 (PMC12722522; doi:10.3389/fimmu.2025.1740279)
Supplement: Supplementary Figure 1 — Downregulation of TICRR inhibits LUAD Progression in A549 cells. A. Verification of TICRR knockdown efficiency by RT-qPCR. B. The cell proliferation of TICRR-deleted A549 cells detected by MTT assay. C. Colony formation assay in TICRR-deleted A549 cells. The cell invasion and migration assay of TICRR-deleted A549 cells with (D) or without Matrigel (E). F. wound healing assays of TICRR-deleted A549 cells. G. EdU assay in TICRR-deleted A549 cells. *p < 0.05, **p < 0.01, ***p < 0.001. [file Table1.docx]

Supplementary file

Table S1. The sequences of primers of TICRR.

| Forward Primer | TGCTGTCACAAAGTAATGCTGC |
| --- | --- |
| Reverse Primer | GCAACTCAGATAGGTGAGGAGG |

Table S2. Oligonucleotides of siRNAs.

| siTICRR-1 | GGAACTTCGTACCAGATCA |
| --- | --- |
| siTICRR-2 | GAAGGTACCTCTCTTGAAA |
| siTICRR-3 | CATGGACAGTCACCCTAGA |
